# American Medical Association

**Published:** 1860-07

**Authors:** 


THE AMERICAN MEDICAL ASSOCIATION.—The thirteenth annual session of this body was held at New Haven on the fifth, sixth, and seventh of June, Dr. Henry Miller presiding, who, on the first day, delivered his valedictory address, which embraced, among other topics, the important one of medical education. The following gentlemen were then elected officers for the ensuing year: *President*, Dr. Eli Ives, of Connecticut; *Vice-Presidents*, Drs. Wilson Jewell, of Pennsylvania, A. B. Palmer, of Michigan, J. P. Logan, of Georgia, and J. N. McDowell of Missouri. *Treasurer*, Dr. Caspar Wistar, of Pennsylvania. On taking his seat, the President made a short address; and, on account of his advanced years, the duty of presiding over the deliberations of the Association devolved upon Dr. Jewell, who executed his task in an admirable manner.

On the second and third days much time was occupied with the discussion of medical education, and a number of resolutions were adopted recommending medical schools to prolong their courses, and institute new chairs. The Committee on Prize Essays reported that they had received no paper worthy of an award. The special committees appointed to report this year on various surgical and medical topics presented, as usual, but few papers, the great majority being either laid over, discharged, or reporting progress; and all the communications were referred to their proper sections. The usual number of standing and special committees were appointed to report on various subjects at the next meeting; and a committee was constituted to collect subscriptions of not more than one dollar each from every regularly educated physician, in aid of the memorial to John Hunter.

Nearly five hundred delegates were present; and the Association was handsomely entertained by the citizens of New Haven. The next meeting will be held in Chicago, on the first Tuesday in June. We regret that our limited space compels us to make so short an abstract of its proceedings.

